# Transcranial Direct Current Stimulation Enhances Muscle Strength of Non-dominant Knee in Healthy Young Males

**DOI:** 10.3389/fphys.2021.788719

**Published:** 2021-12-20

**Authors:** Panpan Lu, Nicholas J. Hanson, Lin Wen, Feng Guo, Xiaoyu Tian

**Affiliations:** ^1^Department of Sports, Nanchang Institute of Technology, Nanchang, China; ^2^Department of Human Performance and Health Education, College of Human Development and Education, Western Michigan University, Kalamazoo, MI, United States; ^3^Graduate School of Biomedical and Health Sciences, Hiroshima University, Hiroshima, Japan; ^4^College of Human Kinesiology, Shenyang Sport University, Shenyang, China; ^5^School of Physical Education, Hainan Normal University, Haikou, China

**Keywords:** transcranial direct current stimulation, surface electromyography, rate of force development, maximal isometric strength, strength performance

## Abstract

Transcranial direct current stimulation (tDCS) has been applied in training and competition, but its effects on physical performance remain largely unknown. This study aimed to observe the effect of tDCS on muscular strength and knee activation. Nineteen healthy young men were subjected to 20 min of real stimulation (2 mA) and sham stimulation (0 mA) over the primary motor cortex (M1) bilaterally on different days. The maximal voluntary contraction (MVC) of the knee extensors and flexors, and surface electromyography (sEMG) of the rectus femoris (RF) and biceps femoris (BF) were recorded before, immediately after, and 30 min after stimulation. MVC, rate of force development (RFD), and sEMG activity were analyzed before and after each condition. MVC of the non-dominant leg extensor and flexor was significantly higher immediately after real stimulation and 30 min after stimulation than before, and MVC of the non-dominant leg flexor was significantly higher 30 min after real stimulation than that after sham stimulation (*P* < 0.05). The RFD of the non-dominant leg extensor and flexor immediately after real stimulation was significantly higher than before stimulation, and the RFD of the non-dominant leg extensor immediately after real stimulation and 30 min after stimulation was significantly higher than that of sham stimulation (*P* < 0.05). EMG analysis showed the root mean square amplitude and mean power frequency (MPF) of the non-dominant BF and RF were significantly higher immediately after real stimulation and 30 min after stimulation than before stimulation, and the MPF of the non-dominant BF EMG was significantly higher 30 min after real stimulation than that after sham stimulation (*P* < 0.05). Bilateral tDCS of the M1 can significantly improve the muscle strength and explosive force of the non-dominant knee extensor and flexor, which might result from increased recruitment of motor units. This effect can last until 30 min after stimulation, but there is no significant effect on the dominant knee.

## Introduction

An overarching goal pursued by sports professionals and scientists is to seek safe and effective ways to improve exercise performance for athletes. Transcranial direct current stimulation (tDCS) is a non-invasive brain stimulation technique that alters cortical excitability *via* a low-intensity direct current (1–2 mA) applied to the scalp, over various regions of the cerebral cortex (Nitsche and Paulus, 2000). It is generally accepted that anodal tDCS enhances cortical excitability by reducing the resting membrane threshold of cortical neurons, while cathodal tDCS decreases neuronal excitability (Nitsche et al., 2003). Synaptic plasticity in the motor cortex is associated with muscular strength and can be modified by tDCS. Some studies have shown that this technique could effectively improve training and increase performance (Huang et al., 2019; Alix-Fages et al., 2020; Vieira et al., 2020; Grosprêtre et al., 2021). Some studies have shown that tDCS does not affect lower limb strength (Montenegro et al., 2015; Maeda et al., 2017; Romero-Arenas et al., 2019); however, this may be related to differences in the chosen electrode configuration or stimulation parameters. In a recent meta-analysis, it was shown that unilateral tDCS was more effective than bilateral tDCS in patients with stroke, while bilateral tDCS was more effective than unilateral tDCS to improve motor learning and motor performance in healthy subjects (Halakoo et al., 2020).

At present, there are few studies on the effect of tDCS on knee muscle strength, and most of these studies have mainly focused on the stimulation of either the motor cortex or the prefrontal cortex, rather than bilateral stimulation (Tanaka et al., 2011; Maeda et al., 2017; Vargas et al., 2017). For example, Tanaka et al. (2011) found that there was a 13.2% increase in knee extensor strength in the hemiparetic side compared to a sham treatment when anodal tDCS (2 mA, 10 min) was delivered to the ipsilateral leg area of the primary motor cortex (M1) in chronic stroke patients.

In addition, there are very few studies focusing on the poststimulation effects of tDCS on knee muscle strength, which has major implications for athletes. It has been reported that 2 mA anodal tDCS delivered for 13 min while an individual is at rest has been shown to increase intracortical facilitation for up to 90 min after the stimulation (Nitsche and Paulus, 2000; Stagg and Nitsche, 2011). In addition, since anodal tDCS has been shown to increase cortical excitability, which would enhance muscle strength (Williams et al., 2013; Lattari et al., 2018; Huang et al., 2019), it is necessary to observe modulation of recruitment of motor units during muscle contractions after tDCS.

The Halo Sport (Halo Neuroscience, San Francisco, CA, United States) is a portable neurostimulation device that consists of a headset similar to conventional headphones. It delivers variable direct current up to 2 mA over the scalp through surface electrodes, or “primers,” which induces changes in bihemispheric M1 (Huang et al., 2019). Halo Sport has been applied in training and competition, but its effects on physical performance remain largely unknown. However, it has been reported that tDCS with the Halo Sport can enhance aspects of sprint cycling ability and cognitive performance in healthy individuals (Huang et al., 2019).

The primary purpose of this study was to examine the effects of bilateral anodal tDCS with the Halo Sport on knee muscle strength and EMG activity and its after-effect. It was hypothesized that Halo Sport would improve the strength of bilateral knee flexors and extensors and have a significant after-stimulation effect.

## Materials and Methods

### Subjects

Nineteen healthy male adults that were not habitual exercisers volunteered to participate in this study (mean age 23.3 ± 2.4 years; height 178.3 ± 6.0 cm; and mass 80.7 ± 13.3 kg). Previous studies have suggested that the effects of tDCS might be different for the sexes, e.g., women might have more prominent tDCS effects than men (Kuo et al., 2006; de Tommaso et al., 2014; Russell et al., 2014; Rudroff et al., 2020). Furthermore, the effects of tDCS might also be influenced to some extent by the menstrual cycle in women (Rudroff et al., 2020). Hence, only male subjects were recruited for the study to avoid potential confounding variables. The subjects were all right leg dominant, and none of the subjects suffered from any neurological history or psychiatric disorder, or had implanted electric devices. Subjects were informed about all aspects of the experiment and all signed written informed consent. The experiment protocol was approved by the Ethics Committee of the Shenyang Sport University and conformed to the Declaration of Helsinki.

### Experimental Design and Procedures

This study employed a single-blinded, randomized, and sham-controlled crossover design to compare the effects of tDCS with sham stimulation over the M1 on knee strength and muscle activation. Subjects were required to visit the lab twice. The two experimental sessions were separated by at least 4–5 days and carried out at the same time of a day to eliminate any circadian effects on strength (Huang et al., 2019; Codella et al., 2020). On the first visit, body mass and height were measured. Subsequently, subjects completed a 15-min warm-up (10 min of light jogging on a treadmill at 8 km/h and static stretching of lower limbs for 5 min) and then a 5 min rest period. At the end of the warm-up, before starting the experiment, subjects performed three maximal voluntary contractions (MVC) with each leg for knee flexors and extensors for the baseline test. For calculating the rate of force development (RFD), subjects were asked to elicit maximal force levels as quickly as possible during the MVC tests. In addition, the EMG signals from the rectus femoris (RF) and biceps femoris (BF) were sampled during the MVC testing. Previous studies have shown that among lower limb muscles, the RF plays a critical role in deceleration and extension, whereas the BF assists the flexion and overall action of the knee joint. The synergy of both muscle groups helps to maintain the balance of internal and external pressure within the knee joint (Yang et al., 2018). Experimental testing consisted of a real tDCS or sham stimulation for 20 min under resting state, the order of which was randomized. Any possible adverse effects of tDCS were recorded after stimulation and the next morning. Immediately and 30 min after the intervention the MVC tests were performed again. During the second visit, the basic testing procedure was the same as the first visit. Data were compared among preintervention (baseline test), directly after, and 30 min after intervention.

### Transcranial Direct Current Stimulation Procedures

**FIGURE 1:**
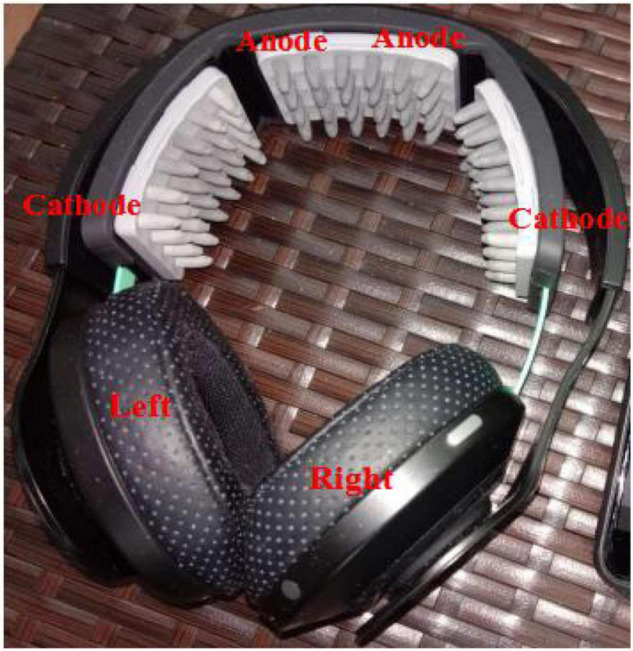
The experimental setup for tDCS (Halo Sport) and the placement of the electrodes (Yang et al., 2018; Codella et al., 2020).

Subjects sat in a comfortable chair in a resting state. The Halo Sport was correctly positioned on the head. To ensure good electrical contact with the scalp, three primers as electrodes were soaked in normal saline (0.9% NaCl) before administration. The size of the electrodes affixed to the scalp was 28 cm^2^ (6.4 cm × 4.4 cm). Referring to the research of Yang et al. (2018) and Codella et al. (2020), the primers were positioned over the center, left, and right leg regions of M1, crossing the vertex of the head. The anode was located at Cz (Jurcak et al., 2007), and the cathodes were approximately located at C5 and C6, therefore bilaterally stimulating the motor cortex (Figure 1). The current was set at 2 mA and the stimulation duration was 20 min. In the active stimulation, the electrical current gradually increased up to 2 mA over 30 s, and thereafter was kept at this level for 20 min. In the sham condition, the electrical current was first ramped up for 30 s, after which it was terminated. This is a reliable method of introducing the initial itching sensation associated with tDCS so that subjects believe that the stimulation is occurring (Palm et al., 2013). Similar stimulation settings have been tested in multiple clinical trials and have proven to be safe in humans (Vines et al., 2008; Kantak et al., 2012).

### Maximal Voluntary Contraction Test, Rate of Force Development, and Evaluation of Dominance of Lower Limb

The MVC testing was performed by a dynamometer system (HUR, Finland). Subjects were asked to sit on a bench with their back firmly fixed into the backrest and hands grasping the handles. The isometric MVC force of the knee flexors and extensors was measured at a 120° knee joint angle (Saito and Akima, 2013). For the MVC test of the knee extensor, subjects extended their knees against a resistance arm as soon as possible to maximal force for 5 s. This process was repeated three times with a rest interval of 30 s. After a 5-min rest, the MVC test of the knee flexor was performed in the same method. After a 5-min rest, the MVC of the other knee was measured. The order of testing (right or left) was randomized. Based on the subject’s MVC, the RFD was calculated according to a reference (Molina and Denadai, 2012). The average values of MVC and RFD for all three trials were calculated and used for further analysis.

In addition, the MVC force level of the knee was used to evaluate the dominance of the lower limb; specifically, the side with a larger knee force was defined as the dominant lower limb and the other was the non-dominant limb (Workman et al., 2020a).

### Surface Electromyography Assessment

The surface electromyography (sEMG) signals of bilateral RF and BF during MVC testing were recorded with a portable sEMG system (Shimmer3 EMG, Shimmer Company, Ireland). Two adhesive surface electrodes were attached over the largest part of the belly of the selected muscle in a bipolar configuration, while the reference electrode was placed over the skin of the tibial tuberosity. The sampling rate of the signals was set at 1,024 Hz and the measured data were converted to digital format *via* a 12-bit analog-to-digital converter. In addition, 8–450 Hz bandpass filter and 49–51 Hz notch filter were employed, in an attempt to minimize the influence of power frequency signal (50 Hz). The root mean square (RMS) and MPF of sEMG of BF and RF during MVC were analyzed. Normalized RMS was adopted in this study, i.e., the RMS value of each stage was divided by the RMS value corresponding to the maximal value of three MVCs during the baseline test. All data were expressed as percentages.

### Statistical Analysis

SPSS 17.0 software and GraphPad Prism 8 were used for statistical analysis and drawing graphs, respectively. The normality of the data distribution was evaluated by Shapiro–Wilk tests. Analyses of MVC, RFD, and sEMG activities of RF and BF during MVC tests were conducted utilizing two-way [condition (two levels: tDCS and sham) × time (three levels: preintervention, immediately, and 30 min after intervention] repeated measures ANOVA. The sphericity was examined using Mauchly’s test, and *post hoc* analysis with LSD was used if significant main or interaction effects were observed. *Post hoc* comparisons were performed using the false discovery rate (FDR) correction. Also, the dominance of the lower limb was assessed using paired sample *t*-tests. In addition, adverse effects of tDCS were compared between groups by χ^2^ test. Effect size (ES) values were denoted by Cohen’s *d* for the pairwise tests, and the criteria to interpret the magnitude of ES were as follows: < 0.2, trivial; 0.2–0.5, small; 0.5–0.8, moderate; and > 0.8, large (Cohen, 1992). All data were expressed by mean ± SE (standard error of the mean) and statistical significance was set *a priori* at *P* < 0.05.

## Results

### Adverse Effects of tDCS

None of the subjects requested the stimulation be discontinued due to discomfort; all subjects completed the testing in full. Adverse effects during each condition are displayed in Table 1. Overall, the stimulation was well tolerated and no serious adverse events were reported. Adverse effects were not significantly different between 2 mA tDCS and sham application (*P* > 0.49).

**TABLE 1:** Comparison of adverse effects between experimental and sham tDCS.

	Experimental (*n* = 19)	Sham (*n* = 19)	*P*
Headache *n* (%)	1 (5.3)	0	1.00
Itching *n* (%)	6 (31.6)	4 (21.1)	0.71
Tingling *n* (%)	9 (47.4)	6 (31.6)	0.51
Burning *n* (%)	2 (10.5)	0	0.49
Dizziness *n* (%)	1 (5.3)	0	1.00

### Determination of Dominant Lower Limb

**FIGURE 2:**
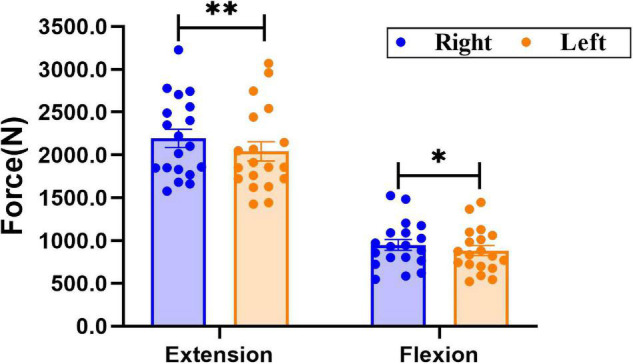
Comparison of bilateral knee extensor and flexor strength. Data are presented as mean ± SEM. Asterisks (*) indicate statistically significant difference (*P < 0.05, **P < 0.01).

As shown in Figure 2, there was a significant difference in knee extensor and flexor strength between sides. The force of the right knee extensor was significantly greater than that of the left knee extensor (Right: 2,193.2 ± 106.7 N; Left: 2,042.8 ± 112.2 N, *P* = 0.006); force in the right knee flexor was significantly larger than that of the left knee flexor (Right: 947.8 ± 62.1 N; Left: 882.2 ± 59.0 N, *P* = 0.025).

### Rate of Force Development

**FIGURE 3:**
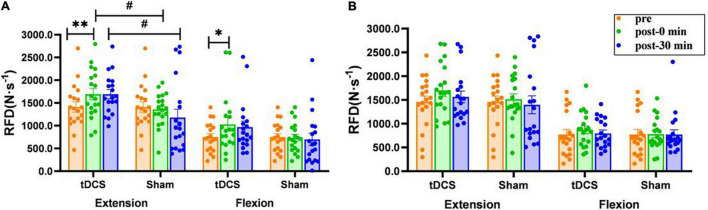
Effects of tDCS on rate of force development (N⋅s^–1^) of left **(A)** and right **(B)** knee flexors and extensors. pre: prestimulation, post-0 min: 0 min after stimulation, post-30 min: 30 min after stimulation. Data are presented as mean ± SEM. Asterisks (*) indicate statistically significant difference compared to prestimulation (*P < 0.05, **P < 0.01). Well number (#) indicates statistically significant difference compared to sham (^#^*P* < 0.05).

There was a significant interaction (condition × time) for the RFD of left leg extension [*F*_(2, 35)_ = 3.952, *P* = 0.028], and a significant main effect of condition was also observed [*F*_(1, 36)_ = 5.220, *P* = 0.028]. The RFD of left leg extension (pre: 1409.4 ± 113.2 N⋅s^–1^; immediate: 1,691.7 ± 122.0. N⋅s^–1^, *P* = 0.008, ES = 0.55) and flexion (pre: 751.8 ± 74.4 N⋅s^–1^; immediate: 1,028.8 ± 146.2 N⋅s^–1^, *P* = 0.019, ES = 0.54) immediately after real stimulation was significantly greater than that before stimulation, and the RFD of left leg extension immediately after (tDCS: 1691.7 ± 122.0. N⋅s^–1^; sham: 1,359.8 ± 82.7 N⋅s^–1^, *P* = 0.031, ES = 0.73) and 30 min after real stimulation (tDCS: 1,690.8 ± 100.7 N⋅s^–1^; sham: 1,178.3 ± 182.9 N⋅s^–1^, *P* = 0.019, ES = 0.79) was significantly greater than that in the sham condition. However, there was no significant interaction, time or condition effect on the RFD of right knee extensor and flexor [Extensor: interaction: *F*_(2, 35)_ = 0.407, *P* = 0.639; condition: *F*_(1, 36)_ = 0.721, *P* = 0.401; time: *F*_(2, 35)_ = 1.022, *P* = 0.357. Flexor: interaction: *F*_(2, 35)_ = 0.165, *P* = 0.847; condition: *F*_(1, 36)_ = 0.189, *P* = 0.667; time: *F*_(2, 35)_ = 0.218, *P* = 0.804, Figure 3)].

### Maximal Voluntary Contraction

**FIGURE 4:**
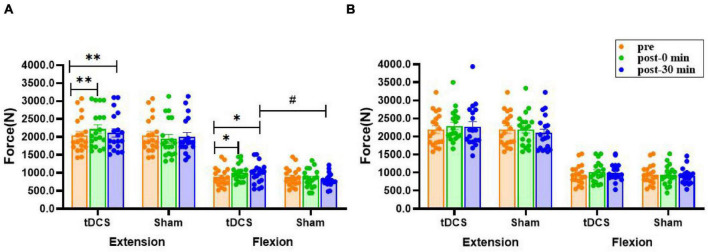
Effects of tDCS on maximal voluntary contraction of left **(A)** and right **(B)** knee flexors and extensors. pre: prestimulation, post-0 min: 0 min after stimulation, post-30 min: 30 min after stimulation. Data are presented as mean ± SEM. Asterisks (*) indicate statistically significant difference compared to prestimulation (*P < 0.05, **P < 0.01). Well number (#) indicates statistically significant difference compared to sham (^#^*P* < 0.05).

There was a significant interaction effect for the MVC of left knee extension [*F*_(2, 35)_ = 9.756, *P*<0.01] and flexion [*F*_(2, 35)_ = 4.109, *P* = 0.021]. Compared to before stimulation, the MVC force of left knee extension (pre: 2,042.8 ± 112.2 N; immediate: 2,219.5 ± 119.2 N, *P* = 0.004, ES = 0.35; 30 min: 2,111.7 ± 118.2 N, *P* = 0.002, ES = 0.13) and flexion (pre: 882.2 ± 59.0 N; immediate: 1,003.7 ± 54.0 N, *P* = 0.012, ES = 0.49; 30 min: 985.0 ± 67.0 N, *P* = 0.049, ES = 0.37) were significantly increased immediately after and 30 min after real stimulation. Moreover, the MVC of left leg flexion 30 min after real stimulation was significantly higher compared to sham tDCS (tDCS: 985.0 ± 67.0 N; sham: 801.1 ± 42.4 N, *P* = 0.026, ES = 0.75). On the other hand, none of the interventions had a prominent effect on right extensor and flexor (*P* > 0.08, Figure 4).

### Root Mean Square

**FIGURE 5:**
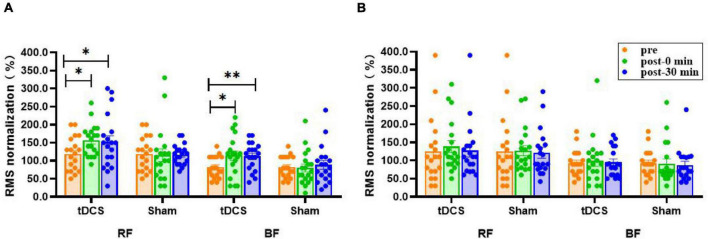
Effects of tDCS on RMS amplitudes (%) of left **(A)** and right **(B)** rectus femoris and biceps femoris sEMG. pre: prestimulation, post-0 min: 0 min after stimulation, post-30 min: 30 min after stimulation; RF: rectus femoris, BF: biceps femoris. Data are presented as mean ± SEM. Asterisks (*) indicate statistically significant difference compared to prestimulation (*P < 0.05, **P < 0.01).

There was no significant interaction (condition × time) for the RMS amplitudes of bilateral RF and BF sEMG (*P* > 0.10), and no main effect of time (*P* > 0.13). However, the main effect of condition was significant for the RMS amplitudes of left RF [*F*_(1, 36)_ = 5.531, *P* = 0.024] and BF [*F*_(1, 36)_ = 4.320, *P* = 0.045] sEMG. The RMS amplitudes of left RF (pre: 118.4 ± 10.7; immediate: 156.3 ± 10.1, *P* = 0.035, ES = 0.83; 30 min: 152.1 ± 17.3, *P* = 0.025, ES = 0.54) and BF (pre: 82.1 ± 6.8; immediate: 114.2 ± 13.1, *P* = 0.032, ES = 0.70; 30 min: 112.1 ± 8.8, *P* = 0.007, ES = 0.87) sEMG immediately after and 30 min after real stimulation were significantly higher compared to before stimulation. Nevertheless, none of the interventions had a prominent effect on right RF and BF (*P* > 0.51, Figure 5).

### Mean Power Frequency

**FIGURE 6:**
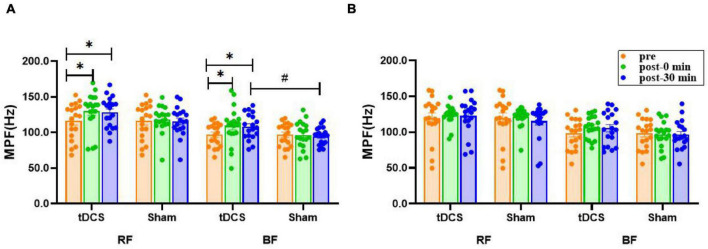
Effects of tDCS on MPF (Hz) of left **(A)** and right **(B)** rectus femoris and biceps femoris sEMG. pre: prestimulation, post-0 min: 0 min after stimulation, post-30 min: 30 min after stimulation; RF: rectus femoris, BF: biceps femoris. Data are presented as mean ± SEM. Asterisks (*) indicate statistically significant difference compared to prestimulation (*P < 0.05). Well number (#) indicates statistically significant difference compared to sham (^#^*P* < 0.05).

There was no significant main effect of time (*P* > 0.17) or interaction (condition × time) (*P* > 0.17) for the mean power frequency (MPF) of bilateral RF and BF sEMG. However, the main effect of condition was significant for left BF [*F*_(1, 36)_ = 5.192, *P* = 0.029]. The MPF values of left RF (pre: 116.2 ± 5.7; immediate: 130.2 ± 6.0, *P* = 0.025, ES = 0.54; 30 min: 128.2 ± 4.9, *P* = 0.047, ES = 0.51) and BF (pre: 97.0 ± 3.7; immediate: 109.6 ± 6.0, *P* = 0.033, ES = 0.57; 30 min: 108.2 ± 4.2, *P* = 0.032, ES = 0.65) sEMG were significantly higher immediately after and 30 min after real stimulation compared to before stimulation, meanwhile, the MPF of left BF sEMG 30 min after real stimulation was increased significantly compared with the sham condition (tDCS: 108.2 ± 4.2; sham: 94.7 ± 2.8, *P* = 0.011, ES = 0.87). No significant difference was noted in right RF and BF (*P* > 0.08, Figure 6).

## Discussion

In many sports such as basketball, boxing, weight lifting, etc., numerous technical movements require athletes to complete rapid muscle contraction in a short period. Explosive force, determined by strength and speed, is the ability of the neuromuscular system to exert maximum muscle strength with maximum acceleration in the shortest time (Girard et al., 2011). The RFD is an important index of explosive force, which is defined as the slope of the force-time curve of muscle under the condition of dynamic and static contraction (Aagaard et al., 2002). It is particularly important to seek effective strategies for athletes to increase their RFD. Athletes with a high RFD have faster muscle contraction speed and can complete motor tasks more quickly, and therefore gain a greater competitive advantage (Baker, 2001).

At present, there are few studies on the enhancement of RFD through tDCS. We found that the RFD of the subjects’ non-dominant leg during extension and flexion increased significantly immediately after 2 mA direct current stimulation over bilateral M1 area; moreover, the RFD of non-dominant leg extension immediately after real stimulation was significantly larger compared with a sham condition. Previous studies have shown that the increase of M1 cortical excitability induced by tDCS can alter the firing frequency of neurons, increase the nerve impulse to muscles, and promote the recruitment of motor units (Dutta et al., 2015). The nerve impulse is an electrical signal sent from the central nervous system to the muscle, which is conducive to driving the recruitment of motor units and triggering the muscle to generate force (Halo Neuroscience, 2016). Some studies have pointed out that RFD is closely related to the recruitment of nerve to motor units per unit time, the frequency of nerve impulses, and the type of muscle contraction (Aagaard et al., 2002). Hence, the enhancement of RFD in this study may be relevant to the increased cortical excitability induced by tDCS. The results of this study are also supported by other published studies. Halo Neuroscience (2016) found that 15 min of 2 mA bihemispheric tDCS administered *via* the Halo Sport device aggrandized vastly the RFD of non-dominant hand in healthy right-handed subjects during an isometric pinch force task. Likewise, Cates et al. (2019) found the healthy right-handed subjects receiving 2 mA of anodal tDCS for 15 min exhibited enhancement in peak rate of force development (pRFD) of non-dominant ballistic thumb during and after stimulation compared to the sham condition. These results indicate that tDCS may contribute to the increases in RFD of the non-dominant limb.

In addition to the immediate effect of tDCS, the after-effect of tDCS on cortical excitability was also observed in this study; namely, the RFD of non-dominant leg extension 30 min after tDCS intervention was significantly greater than that of the sham condition. Tanaka et al. (2009) observed that following 10 min of anodal tDCS at 2 mA over the contralateral leg motor cortex, the maximal pinch force of the left leg in healthy adult subjects was transiently enhanced, and the augment effect lasted for 30 min after the end of tDCS sessions. The results of this study are consistent with these studies, meaning that tDCS may have significant after-effects. However, our study found that the RFD improved 30 min after real stimulation, but not as significantly as in the immediate period after stimulation. A previous study found the after-effects of tDCS increasingly decreased with the prolongation of time after stimulation (Mashal and Metzuyanim-Gorelick, 2019). The duration of the after-effects is dependent on stimulation intensity, duration, and times (Nitsche and Paulus, 2001). It is reported that the stimulation effect can last for 30–60 min following a single tDCS session for 10–20 min (Ardolino et al., 2005). The repetitive intervention of more than 1 week is capable of having an effect for 1–2 weeks (Butts et al., 2014) and the after-effects of prolonged tDCS can even be detected a few months later (Reis et al., 2009). Regarding current intensities, a recent study reported that the intensity of 2 mA may not be sufficient to affect neuronal circuits (Holgado et al., 2019). Vöröslakos et al. (2018) proposed by testing transcranial alternating current stimulation (tACS) that because a large part of the current is lost as a result of soft tissues, skin, and resistance of the skull, at least 4.5 mA would be required to affect the neuronal circuits. Nevertheless, there is a relative paucity of data on the effectiveness and safety of higher current intensity on exercise capacity in healthy populations at present (Bikson et al., 2016; Esmaeilpour et al., 2018). Recently, Workman et al. (2020b) found 4 mA tDCS was generally well tolerated by the young, healthy right-side dominant subjects; however, the 2 and 4 mA tDCS intensities increased the fatigability of the right knee extensors during an isokinetic fatigue test, which might be caused by changes in motor unit recruitment/discharge rate or cortical hyperexcitability. Similarly, Workman et al. (2020a) found in another study that 4 mA tDCS over the left M1 was well-tolerated and but also increased the fatigability of the left knee flexors in young, healthy right-side dominant subjects. Therefore, the practice of applying high-intensity electrical stimulation in an attempt to obtain the gain effects needs to be carefully considered. Furthermore, as a result of the high variability among individuals, the most effective measure may be to apply individualized current intensity to the subjects (Vöröslakos et al., 2018).

We found that tDCS increased the RFD, and its after-effects could last for 30 min, which might be explained by an increased firing rate of previously recruited motor units. These findings demonstrate that tDCS delivered *via* the Halo Sport may be a safe and effective method to facilitate explosive strength for healthy populations in daily life or exercise training.

It is generally believed that motor unit recruitment strategy is crucial in the process of maximum force generation (Farina and Negro, 2015). Previous studies have indicated that motor unit recruitment and synchronicity can be modulated by anodal tDCS (Dutta et al., 2015). As a consequence, it can be inferred that this neuromodulatory technique may contribute to the improvement of the MVC force. We found that the MVC of the non-dominant flexor and extensor groups immediately after and 30 min after real stimulation increased significantly compared with before stimulation; furthermore, the MVC of the non-dominant leg during flexion 30 min after tDCS intervention was greater than the sham condition. The results of this study are consistent with the results reported in previous studies. For instance, Washabaugh et al. (2016) found that 2 mA, 12 min of anodal tDCS vastly aggrandized the knee extensor torque and MVC ability in healthy subjects than sham stimulation, and the stimulation effect was also remarkable 25 min after stimulation. A plausible explanation for the improvement in muscular strength is that tDCS-induced changes in corticospinal excitability increase the recruitment of motor units, thus leading to greater muscle strength during contraction (Krishnan et al., 2014).

Interestingly, the pronounced effect of the lower limb muscle strength enhancement after tDCS administration was mainly manifested in the non-dominant leg. Muscle strength of the dominant leg did show an upward trend after stimulation, but with no statistical difference compared to the sham. Asymmetric use of the dominant and non-dominant legs might elicit asymmetry of cortical excitability between the dominant and non-dominant hemisphere, namely, the excitability of the non-dominant motor cortex is lower than that of the dominant motor cortex (Boggio et al., 2006). Boggio et al. (2006) investigated the effect of anodal tDCS of the dominant and non-dominant M1 on the hand motor function in healthy right-handed subjects. Their results showed that non-dominant hand (left-hand) motor function was significantly improved by anodal stimulation (1 mA, 20 min), whereas neither anodal nor sham tDCS gave rise to a prominent change in the dominant hand motor performance. The possible reason for the lack of effects on the dominant hemisphere is that there may be a ceiling effect on the stimulation effect of tDCS on the dominant side. Since the cerebral dominant hemisphere is already optimally activated, an additional increase in excitability by anodal tDCS would not provide further behavioral benefits to these subjects (Boggio et al., 2006). Nonetheless, in a study by Vargas, 20 adolescent female soccer players underwent five MVC tests of bilateral knee extensors after 2 mA anodal tDCS and found significant improvement in MVC force in the dominant limb (Vargas et al., 2017). These inconsistent findings suggest that further studies are needed to confirm whether there is a ceiling effect of tDCS on the enhancement of athletic abilities. Although the effects of tDCS on the dominant limb muscle strength are controversial, the results of this study suggest that tDCS has the potential to augment the muscle strength of non-dominant limbs. In future experimental research and actual sports training, tDCS technology can be employed to increase the non-dominant limb muscle strength of athletes, narrow the gap with the dominant limb, avoid the imbalance phenomenon, and further boost the overall exercise capacity, which would have an exceedingly practical significance for muscle strength training and the prevention of sports injury.

The main mechanism of enhancing muscular strength by tDCS is to regulate the nerve factors related to muscle strength (Ardolino et al., 2005). EMG is capable of reflecting the influence of nerve drive and other factors during muscle contraction (Enoka, 2011). Fast-twitch (type II) muscle fibers are commonly innervated by high-threshold neurons. These muscle fiber types are usually closer to the surface, and their contractile changes can be well recorded *via* sEMG signals (Williams et al., 2013). The changes in motor unit recruitment, in a sense, can be traced by sEMG (Zhou and Rymer, 2004). A previous study has argued that the peak torque and EMG amplitude of biceps brachii in healthy right-handed young adults during maximum contraction can be significantly increased after 2 mA, 10 min anodal tDCS over the left M1. The author believes that muscle activation may be related to the changes in motor unit recruitment strategies (Krishnan et al., 2014). The results of this study showed that the activation level of non-dominant BF and RF was significantly higher immediately after and 30 min after bilateral tDCS over M1 than before. Nevertheless, Kan et al. (2013) found that 2 mA of anodal tDCS applied to right M1 for 10 min did not affect the RMS amplitude of non-dominant biceps brachii in an isometric (30% MVC) muscle endurance test of the elbow flexors in healthy male subjects. The inconsistency of previous results may be related to the experimental scheme, electrode configuration, etc. In the study of Kan et al., electrodes were placed in unilateral cerebral motor areas (right M1), while bilateral tDCS over M1 was used in this study. Recent studies have been reported that tDCS at bilateral brain motor areas could elevate muscle power in the lower limb (Yang et al., 2018; Huang et al., 2019). Yang et al. (2018) found that the group receiving 2 mA of stimulation for 20 min targeting the motor cortex bilaterally of tDCS with Halo Sport device was more effective in improving balance ability and muscle activation of the RF and BF than action observation training group. Huang et al. (2019) also observed a performance benefit from the Halo Sport, specifically improving repeated sprint cycling power output. They suggest one possible mechanism is that the stimulation elicits increases in intracortical facilitation and motor cortex excitability, promoting connections between neurons in the motor cortex. This may enhance the motor drive to the muscles, thus increasing power output. Consequently, bilateral tDCS over the M1 may modulate central nervous system function and improve power and power output.

Furthermore, no significant change was noted in the activation level of the dominant flexor and extensor muscles after tDCS treatment, which was consistent with the results of muscle strength in this study. Some studies have determined that tDCS will not further heighten muscle function after it has reached a maximum level (Lattari et al., 2016; Abdelmoula et al., 2019). Considering that the subjects selected in this study were all right leg dominant, the muscle activation level has reached the best state in high-intensity exercise, and in this state, the ability of tDCS to increase the number of motor unit recruitment to improve the muscle activation degree might be limited, resulting in no significant improvement in muscle strength performance.

There is a complex relationship (linear and non-linear correlation) between sEMG amplitude and muscle strength; generally speaking, there is a linear correlation between sEMG amplitude and muscle force at lower force levels. However, the correlation may be non-linear at higher force levels, e.g., the sEMG amplitude may increase exponentially as the muscle force increases (Felici et al., 2001). Several studies point out, however, that the changes of sEMG amplitude and power spectrum are not only related to muscle strength but also related to fatigue degree (Luttmann et al., 2000). The time-domain EMG signal tends to increase with the enhancement of muscle strength and the generation of fatigue, while the frequency-domain EMG signal increases with the improvement of muscle strength, but decreases with the generation of fatigue (Luttmann et al., 2000). Consequently, MPF was employed in an attempt to determine whether the increase of RMS value is caused by the augment of muscle strength. Our results showed that the MPF values of non-dominant RF and BF sEMG were significantly higher immediately after and 30 min after real stimulation compared to before stimulation, meanwhile, the MPF of non-dominant BF sEMG was increased significantly 30 min after real stimulation compared with the sham condition. Also, the corresponding MVC values immediately after and 30 min after tDCS intervention were increased, so the increase of RMS values could exclude the effects of fatigue factors (Luttmann et al., 2000). The changes of EMG amplitude are related to the number of motor unit recruitment (Frazer et al., 2017) and impulse frequency (Komi et al., 2000). Therefore, tDCS was likely to promote the recruitment of motor units, increase the level of muscle activation, and then enhance muscle strength in this study.

While this study revealed that bihemispheric tDCS over M1 using the Halo Neurostimulation System could facilitate the strength performance and explosive force of knee joint to some extent, several limitations should be noted. First, the present research was restricted to normal healthy young male subjects. Future studies should utilize athletes as subjects; this can provide a reliable basis for tDCS technology to serve the realm of athletics better. In addition, this study used a single tDCS session; repeated studies should be conducted to evaluate the long-term stimulation effect of tDCS in the future. There may be sex-related differences in the response of subjects to tDCS; specifically, the motor performance of male and female subjects may be different (Russell et al., 2014). Hence, future research should seek to enroll both sexes. At last, neuroimaging techniques such as electroencephalography can be utilized to explore the mechanism of tDCS in improving motor performance.

## Conclusion

A single tDCS session bilaterally over the M1 can significantly improve the muscle strength and explosive force of the non-dominant knee, which might result from increased recruitment of motor units. The effect on muscle strength can last until 30 min after stimulation, but there is no significant effect on the dominant knee.

## Data Availability Statement

The original contributions presented in the study are included in the article/supplementary material, further inquiries can be directed to the corresponding author/s.

## Ethics Statement

The studies involving human participants were reviewed and approved by Shenyang Sports University; Shenyang, Liaoning, China. The patients/participants provided their written informed consent to participate in this study.

## Author Contributions

PL carried out the experiments and wrote the manuscript. XT designed the experiments. NH analyzed the data and revised the manuscript. LW participated in the recording, processing, and analysis of the data. FG designed the study, revised and wrote the original manuscript. All authors read and approved the final manuscript.

## Conflict of Interest

The authors declare that the research was conducted in the absence of any commercial or financial relationships that could be construed as a potential conflict of interest.

## Publisher’s Note

All claims expressed in this article are solely those of the authors and do not necessarily represent those of their affiliated organizations, or those of the publisher, the editors and the reviewers. Any product that may be evaluated in this article, or claim that may be made by its manufacturer, is not guaranteed or endorsed by the publisher.

## Abbreviations

tDCS: Transcranial direct current stimulation
RFD: Rate of force development
MVC: Maximal voluntary contraction
sEMG: Surface electromyography
RMS: Root mean square
MPF: Mean power frequency
RF: Rectus femoris
BF: Biceps femoris
M1: Primary motor cortex.
